# The heritability of coronal and sagittal phenotype in idiopathic scoliosis: a report of 12 monozygotic twin pairs

**DOI:** 10.1007/s43390-020-00172-8

**Published:** 2020-08-06

**Authors:** Tom P. C. Schlösser, Ane Simony, Paul Gerdhem, Mikkel Ø. Andersen, René M. Castelein, Diederik H. R. Kempen

**Affiliations:** 1 Department of Orthopaedic Surgery, University Medical Center Utrecht, Postbus 85500, Utrecht, The Netherlands; 2 Center for Spine Surgery & Research, Middelfart Hospital, Middelfart, Denmark; 3 Department of Reconstructive Orthopaedics, Karolinska Institutet, Institute of Regional Health Research, Karolinska University Hospital and CLINTEC, Stockholm, Sweden; 4 University of Southern Denmark, Odense, Denmark; 5 Department of Orthopaedic Surgery, OLVG, Amsterdam, The Netherlands

**Keywords:** Phenotype, Genotype, Monozygotic twins, Idiopathic scoliosis

## Abstract

**Purpose:**

One of the pathways through which genetics may act in the causation of idiopathic scoliosis is inheritance of a specific sagittal profile that predisposes for its development. In this study, coronal and sagittal parameters were compared in an international collection of monozygotic twins with idiopathic scoliosis.

**Methods:**

Twelve monozygotic twin pairs who underwent biplanar radiography for idiopathic scoliosis were systematically identified in existing scoliosis databases in The Netherlands, Sweden, and Denmark. On the first available radiographs, the coronal and sagittal curve parameters (Roussouly and Abelin types, thoracic kyphosis, lumbar lordosis and length of the posteriorly inclined segment) were determined.

**Results:**

In all 12 monozygotic twin pairs, both twins were affected by AIS. Four (33%) twin pairs had similar coronal and sagittal spinal phenotype, whereas two (17%) had different coronal phenotype and similar sagittal profiles, and six (50%) pairs had different coronal as well as sagittal phenotype.

**Conclusions:**

Analysis of biplanar curve characteristics in monozygotic twins showed that all twin pairs were affected by idiopathic scoliosis. However, only 33% of the pairs had similar coronal and sagittal spinal phenotypes. Based on this limited dataset, the hypothesis can be formulated that besides genetic pre-disposition, the individual (inherited) sagittal profile plays a role in the development of different coronal curve type.

## Introduction

After many years of dedicated research into the etiopathogenesis of idiopathic scoliosis, the number of theories is overwhelming [1–3]. During the last decade, multiple studies found evidence that genetic factors as well as intrinsic spino-pelvic biomechanics of the upright human spine play a role in the causation of idiopathic scoliosis [3–6].

A monozygotic twin study can help to reveal the importance of environmental and genetic influences on the etiology of idiopathic scoliosis. Although monozygotic twins are often exposed to the same environmental factors, they share 100% of their genome which help to identify genetic factors when phenotypical concordance rates are high in pairs. Phenotypic differences, on the other hand, such as the presence of (a certain type of) scoliosis in only one member of a monozygotic pair or different curve types, shifts the evidence towards a more important environmental causation.

To date, several monozygotic and dizygotic twin studies demonstrated heritability of idiopathic scoliosis. So far, these studies mainly focused on the coronal characteristics and little is known on inheritance of the sagittal profile in cases with idiopathic scoliosis [7–9]. Previously, it has been shown that sagittal profile is correlated with the development of different types of idiopathic scoliosis [6]. The aim of this study was to analyze the concordance of sagittal and coronal phenotype in monozygotic twins with idiopathic scoliosis.

## Subjects and methods

### Population

Appropriate use criteria for case series were applied.[10] In the existing scoliosis databases from two scoliosis centers in The Netherlands, one in Sweden and two in Denmark, we systematically searched for monozygotic twin pairs. Zygosity was extracted from medical charts. Pairs in which at least one twin was affected by idiopathic scoliosis and both had undergone biplanar (posterior–anterior and lateral) radiographic evaluation of the full spine were included. Cases with non-idiopathic scoliosis or other spinal abnormalities were excluded. The first PA and lateral radiographs of each twin pair, acquired less than 3 months apart, were extracted from the databases. Original analog radiographs were digitized prior to measurement of the outcome parameters.

### Measurement of coronal and sagittal phenotype

In the coronal plane, on the posterior–anterior radiographs, Cobb angles were measured.[11] Since bending radiographs were not available on all cases, curves were classified according to the Lenke curve types.[12] Similarity of coronal curve morphology was defined as an identical coronal curve type. On the lateral radiographs, sagittal phenotype was classified according to Roussouly and the recently validated Abelin-Genevois type for sagittal spinal morphology [13–15]. The Abelin-Genevois classification differentiates between (type 1) normal sagittal alignment, (type 2a) thoracic hypokyphosis, (type 2b) thoracic hypokyphosis + thoracolumbar kyphosis, and (type 3) cervicothoracic kyphosis. Also standard sagittal parameters [pelvic incidence, thoracic kyphosis T4–T12 (TK), lumbar lordosis L1-S1 (LL), and length of the posteriorly inclined segment] were measured by three trained observers using a previously validated image processing technique [16]. The sample size of this case series was considered hypothesis-generating and too small for further statistical analyses.

## Results

**Fig. 1:**
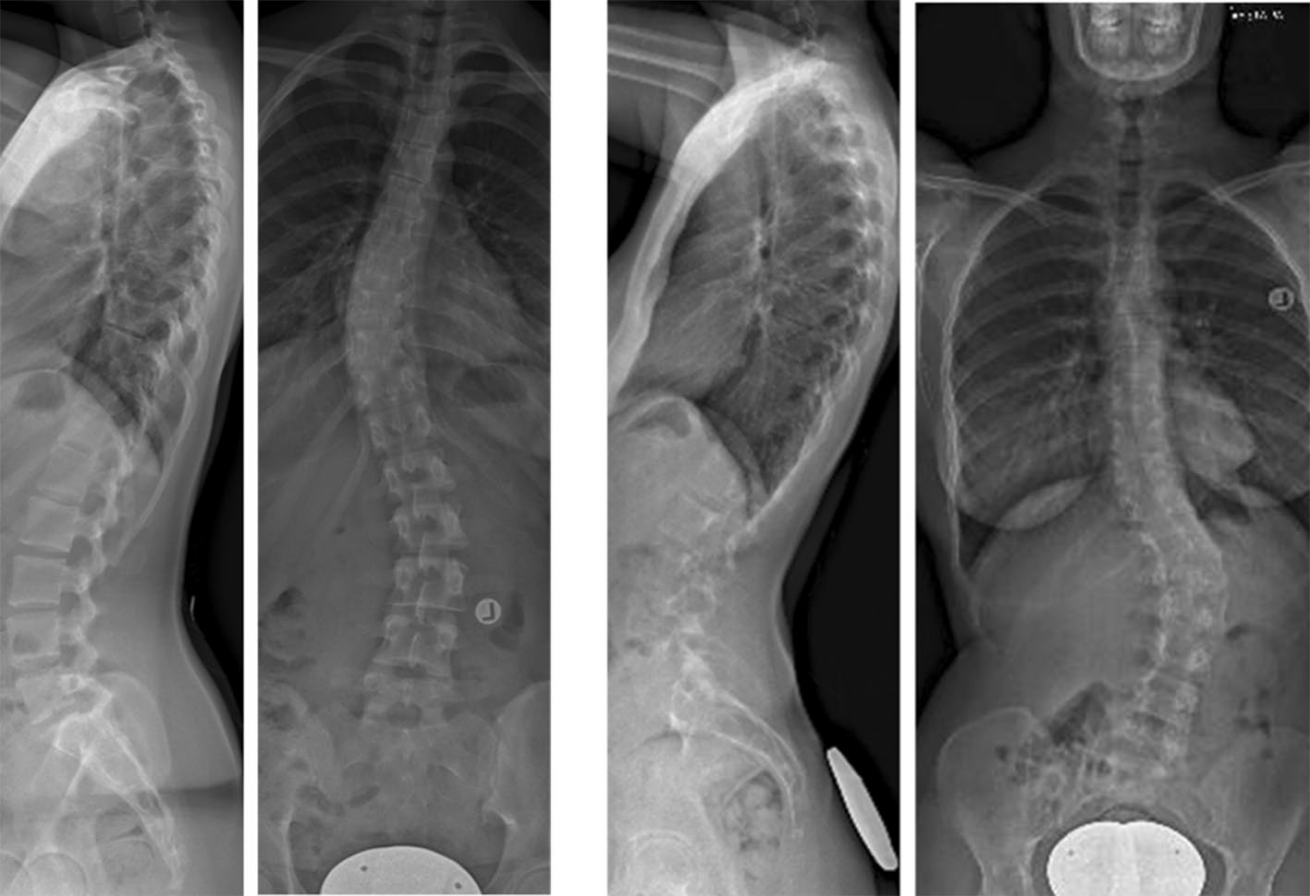
PA and lateral radiographs of a monozygotic twin pair. Radiographs were taken less than 3 months apart. This monozygotic twin pair was characterized by two different coronal curvatures (thoracic vs. thoracolumbar) also had two different sagittal profiles [small versus high pelvic incidence, Abelin-Genevois curve type 2a (hypokyphosis) versus 1 (normokyphosis)]

Twelve monozygotic twin pairs with idiopathic scoliosis were identified. Demographics are shown in Table 1. In all 12 twin pairs, both twins were affected by AIS (concordance of 100%): six (50%) pairs had similar coronal and also a similar sagittal curve pattern. Five (42%) pairs had a different coronal and also a different sagittal phenotype (Fig. 1). One pair (8%) had a similar coronal and a different sagittal pattern. There were no twin pairs with a similar sagittal pattern and different coronal pattern. Sagittal and coronal parameters per twin are shown in Table 2. There was 58% concordance of coronal and 50% of sagittal phenotypes (Table 3).

**Table 1:** Demographics

Twin pair	Gender	Age (years)	Concordancy idiopathic scoliosis
Pair 1	♀	12	Yes
Pair 2	♀	13	Yes
Pair 3	♀	15	Yes
Pair 4	♀	18	Yes
Pair 5	♀	14	Yes
Pair 6	♀	5	Yes
Pair 7	♂	15	Yes
Pair 8	♀	18	Yes
Pair 9	♀	17	Yes
Pair 10	♀	16	Yes
Pair 11	♂	11	Yes
Pair 12	♂	13	Yes

**Table 2:** Sagittal and coronal outcome parameters are presented

Pair twin	Lenke type	Major Cobb (°)	Similar coronal curve morphology?	TK (°)	LL (°)	DL (%)	Roussouly type	Abelin-Genevois type
1-1	1	38	No	17	26	56	2	2a
1-2	5	40		18	31	64	3	2a
2-1	1	15	Yes	31	35	74	3	1
2-2	1	13		36	27	68	3	1
3-1	1	30	No	41	64	52	4	1
3-2	5	32		32	48	56	3	2a
4-1	5	22	No	33	46	65	3	2a
4-2	1	23		27	37	59	4	2b
5-1	1	19	Yes	21	48	69	2	2a
5-2	1	20		18	23	79	2	2a
6-1	1	18	Yes	11	44	32	3	2a
6-2	1	30		1	34	39	3	2a
7-1	5	20	Yes	30	70	68	4	1
7-2	5	43		25	43	70	4	1
8-1	1	34	No	28	57	68	2	1
8-2	5	42		37	67	88	4	1
9-1	5	34	Yes	28	75	84	2	1
9-2	5	35		20	39	76	2	1
10-1	5	26	Yes	27	37	77	3	2a
10-2	5	29		26	58	73	3	2a
11-1	5	11	No	28	39	71	2	1
11-2	1	33		18	40	79	3	2a
12-1	1	44	Yes	30	26	56	1	1
12-2	1	17		22	32	70	2	2a

*TK* thoracic kyphosis, *LL* lumbar lordosis, *DL* length of the posteriorly inclined segment of the spine, relative to total spinal length

**Table 3:** Relation between sagittal spinal alignment and coronal curve type in 12 monozygotic twin pairs

Sagittal phenotype	Coronal phenotype	
	Similar	Different
Similar	6 (50%)	0 (0%)
Different	1 (8%)	5 (42%)

## Discussion

This small monozygotic twin study helps to reveal the importance of environmental and genetic influences on the etiology of different types of idiopathic scoliosis. On one hand, the presence of scoliosis in both members of all monozygotic pairs in this study confirms a genetic causation. On the other hand, the relatively low concordance of coronal and sagittal phenotypic idiopathic scoliosis characteristics shifts the evidence towards genetic pre-disposition and a more important environmental causation for the onset of different curve types.

Previously, larger twin studies on scoliosis showed a strong genetic effect with concordances in monozygotic pairs of 0.72 to 0.92 [17, 18]. Recent population-based studies from the Danish and Swedish twin registries, however, showed lower pairwise concordances of 0.11 to 0.4 in monozygotic twins [8, 9]. From the Swedish mono- and dizygotic twin registry, the variance in the probability to develop scoliosis due to additional genetic effects (heritability) was estimated to be 38% [8]. The selection of the present series of twelve monozygotic twins was biased by the necessity of having biplanar radiographs for both twins, and therefore, there is likely an ascertainment bias. In all likelihood, biplanar radiographs of non-scoliotic twins were not acquired for screening purposes, nor will the ‘normal’ sibling of a scoliotic patient have undergone radiography.

One of the pathways through which genetics in idiopathic scoliosis may act is a specific (inherited) sagittal profile that predisposes for its development [19]. In this study, the phenotypic similarity of global coronal and sagittal shape was compared in an international collection of monozygotic twins with idiopathic scoliosis. The previous studies did not investigate similarity in phenotypic coronal and sagittal curve patterns, but classified concordant as both twins having a scoliosis and discordant when only one had a scoliotic curve. In the 12 concordant monzygotic twins in this report, six pairs (50%) had full agreement on sagittal as well as coronal spinal deformation, whereas five pairs (42%) had a difference in coronal as well as sagittal phenotypical presentation. While some single-gene inheritance disorders such as achondroplasia and Duchenne muscular dystrophy show little variation in phenotypical presentation, even in single-gene disorders, highly concordant phenotypes are rare. In those, it is more the susceptibility to develop the disorder that is inherited. Therefore, the findings of the present study suggest a strong genetic pre-disposition for the initiation of a scoliosis, but, in the meantime, shift the evidence from a genetic towards a more important non-genetic/environmental causation for the development of different curve types.

Recently, it has been shown that the sagittal profile plays an important role in the development of different (phenotypical) types of idiopathic scoliosis [6]. Thoracic and lumbar idiopathic scoliosis were shown to develop on an essentially different sagittal profile as compared to non-scoliotic controls [6]. Also, it was shown that the different sagittal Abelin-Genevois types are already present in very early stages of the development of the scoliosis [20]. The rotational stiffness of spinal segments was decreased by the inclination of individual vertebrae in the sagittal plane as determined by the individual’s inherited sagittal spinal profile, due to posteriorly directed shear loads [21]. Moreover, a previous study has also shown a certain inheritance of sagittal spino-pelvic alignment from parents to the child in adolescent idiopathic scoliosis [19]. Accordingly, in this study, 50% of the twin pairs showed agreement on sagittal phenotype as well as subsequent coronal curve morphology, whereas 42% of the pairs had no agreement on sagittal and coronal phenotype. Therefore, it can be hypothesized that twins affected by idiopathic scoliosis during puberty have a certain genetic profile making them prone to develop a spinal deformity, and that (genetic or environmental) differences in phenotype of sagittal profile may play a role in the development of different coronal curve types. It could be the same genotype with different exposure to environmental factors or different physical characteristics (such as BMI) during childhood in the two twins, causing the different sagittal profile [22, 23]. To obtain further insight in the inheritance pattern of sagittal alignment and onset of different coronal curve types, these should be further explored from parents to the child in a large-scale setting.

## Conclusions

In accordance with the existing literature, that all monozygotic twins in this series were affected by idiopathic scoliosis suggests that genetic factors play a role in the pathogenesis of idiopathic scoliosis. They often have, however, different coronal or sagittal spinal curvatures. Because of the 75% agreement on (dis)similarity in sagittal alignment and coronal phenotype in the 12 pairs of monozygotic twins, it can also be hypothesized that (inheritability of) the sagittal alignment plays a role in the development of different coronal curve types.
